# Association between comorbidity and health-related quality of life in a hypertensive population: a hospital-based study in Bangladesh

**DOI:** 10.1186/s12889-022-12562-w

**Published:** 2022-01-26

**Authors:** Adnan Mannan, Kazi Mahmuda Akter, Farhana Akter, Naim Uddin Hasan A Chy, Nazmul Alam, Susmita Dey Pinky, Abul Faisal Md. Nuruddin Chowdhury, Parijat Biswas, Afrin Sultana Chowdhury, Mohammed Akram Hossain, Md. Mashud Rana

**Affiliations:** 1 Department of Genetic Engineering & Biotechnology, Faculty of Biological Sciences, University of Chittagong, Chattogram, 4331 Bangladesh; 2 Department of Obstetrics and Gynaecology, Sir Salimullah Medical College Mitford Hospital, Dhaka, 1206 Bangladesh; 3 Department of Endocrinology, Chittagong Medical College, Chattogram, 4203 Bangladesh; 4 Health Economics Research Group, Department of Economics, University of Chittagong, Chattogram, 4331 Bangladesh; 5 Department of Public Health, Asian University for Women, Chattogram, 4000 Bangladesh; 6 Department of Medicine, Chittagong Medical College, Chattogram, 4203 Bangladesh; 7 Department of Biotechnology and Genetic Engineering, Noakhali Science and Technology University, Noakhali, 3814 Bangladesh; 8 Department of Cardiology, 250 Bedded General Hospital, Chattogram, 4000 Bangladesh; 9 Department of Pharmacology and Therapeutics, Chittagong Medical College, 4203 Chattogram, Bangladesh

**Keywords:** Bangladesh, Hypertension (HTN), Health-Related Quality of Life (HRQoL), EQ-5D-3L, Comorbidity

## Abstract

**Background:**

Hypertension is a known risk factor for several chronic conditions including diabetes and cardiovascular diseases. However, little is known about its impact on Health-related quality of life (HRQoL) in the context of Bangladesh. This study aimed to evaluate the association of hypertension on HRQoL among Bangladeshi patients corresponding to the socio-demographic condition, comorbid conditions, treatment, and health outcomes.

**Methods:**

A hospital based cross-sectional study was conducted using a pre-tested structured questionnaire among patients with hypertension in 22 tertiary medical college hospitals in Bangladesh. The study recruited male and female hypertensive patients of age ≥18 years between July 2020 to February 2021 using consecutive sampling methods. Health related quality of life was measured using the widely-used index of EQ-5D that considers 243 different health-related attributes and uses a scale in which 0 indicates a health state equivalent to death and 1 indicates perfect health status. The five dimensions of the quality index included mobility, self-care, usual activities, pain or discomfort, and anxiety or depression. Ordered logit regression and linear regression models were used to estimate the predictors of comorbidity and HRQoL.

**Results:**

Of the 1,912 hypertensive patients, 56.2% were female, 86.5% were married, 70.7% were either overweight or obese, 67.6% had a family history of hypertension, and 85.5% were on anti-hypertensive medication. Among the individuals with comorbidities, 47.6% had diabetes, 32.3% were obese, 16.2% had heart disease, 15% were visually impaired, and 13.8% were suffering from psychological diseases. HRQoL was found to be inversely proportional to the number of comorbidities. The most frequent comorbidities of diabetes and obesity showed the highest EQ- 5D mean utilities of 0.59 and 0.64, respectively.

**Conclusions:**

Prevalent comorbidities, diabetes and obesity were found to be the significant underlying causes of declining HRQoL. It is recommended that the comorbidities should be adequately addressed for better HRQoL. Special attention should be given to address mental health issues of patients with hypertension.

**Supplementary Information:**

The online version contains supplementary material available at 10.1186/s12889-022-12562-w.

## Background

With increasing life expectancy and a rise in the aging population, a large number of people are currently living with various chronic diseases worldwide which grossly impairs their Health Related Quality of Life (HRQoL) [1]. Hypertension is a major risk factor for many cardiovascular, cerebrovascular, and renal diseases, which is emerging globally due to lifestyle changes and other factors [2]. In 2010, around 31.1% of adults worldwide had hypertension, whereas, in 2015, more than 1 billion adults were living with hypertension. Maximum of the cases originated from low-and middle-income countries (LMICs) [3, 4]. In Bangladesh, hypertension is one of the ten leading reported causes of death. One out of five Bangladeshi adults have hypertension and approximately 4% of deaths in Bangladesh occur due to complications related to hypertensive disorders [5, 6]. According to a report of World Health Organization (WHO) published in 2018, the prevalence of hypertension in Bangladesh is 21% which is gradually becoming a medical and public health concern for Bangladesh [7].

Health-related quality of life (HRQoL) is a multidimensional archetype, which contains five broad domains: resilience, health perception, functional states, impairment/diseases, and duration of life that altogether denotes the concordance between how long and how well an individual might live [8]. HRQoL assesses the impact of health on one’s ability to live a life of fulfillment in terms of physical, mental, emotional, and social functioning that is reported by individuals themselves [9]. It goes beyond calculating life expectancy, death rate, and causes of death; rather it is related to the impact of health status on overall HRQoL. Given the overgrowing prevalence of hypertension, studying the HRQoL in patients with hypertension has become a necessity, as a measure to determine the quality of care and to guide medical and personal healthcare practices. Although hypertension of mild to moderate grade often remains asymptomatic, other comorbid diseases, complications, and in some cases the adverse effects of anti-hypertensive drugs for long term use may negatively impact the health related quality of life [10].

Previously only a few studies have explored the association between HRQoL and hypertension globally. Among them, a study conducted among the US Hispanic people reported lower HRQoL among the hypertensive patients [11]. HRQoL of hypertensive versus non-hypertensive individuals in Brazil reported that hypertension impaired the HRQoL of patients who suffered from it [12]. Likewise, a study in the Swedish population concluded that people with hypertension had lower perceived levels of HRQoL than normotensive individuals [13, 14]. Some studies, however, have shown that hypertension itself seems to cause less deterioration in HRQoL when the contribution of other comorbidities are taken into consideration [1].

Although some previous studies have evaluated HRQoL among patients of type-2 Diabetes Mellitus in Bangladesh [15–17], similar studies on patients with hypertension are yet to be conducted. Furthermore, in terms of measuring HRQoL, most of the studies used some generic questionnaires, such as SF-36 and WHOQOL-100. Only a few studies administered the EQ-5D-3L questionnaire for evaluating HRQoL [15, 18]. No nationwide study has been carried out among Bangladesh’s hypertensive patients using the EQ-5D-3L version to measure the HRQoL to date.

The objective of this study is two-fold. First, it examined the relationship between hypertension and HRQoL among patients with hypertension taking socio-demographic factors and comorbidities under consideration. Second, it explored the role of comorbidities such as diabetes, chronic obstructive pulmonary disease (COPD), obesity and anxiety/depression on the HRQoL of people with hypertension.

## Methodology

### Study design, setting and participants

This cross-sectional study was carried out among patients with hypertension attending 22 tertiary medical college hospitals of Bangladesh between July 2020 and February 2021. All of these hospitals combinedly provide treatment for around 20 million residents in major cities and adjacent districts. Both men and women aged above 18 years old were included in this study. All the participants were confirmed to have hypertension, diagnosed by medical professionals as defined per WHO criteria of persistent systolic blood pressure (SBP) of ≥140 mm Hg or diastolic blood pressure of ≥90 mm Hg [19]. Besides, individuals who were taking blood pressure-lowering drugs and had the blood pressure within a normal level were also considered hypertensive. Convenience sampling method was used for recruitment of study subjects rigorously following the study inclusion criteria.

### Data collection and variables

#### Data collection

A structured pilot-tested questionnaire was used for data collection. Face-to-face interviews were conducted by trained and qualified data collectors of both male and female sex. The study’s principal investigator and co-principal investigators strictly monitored data collection phases to ensure work efficiency and quality of collected data were maintained. The questionnaire asked for information related to age, sex, education, occupation, monthly income, duration of hypertension, family history of hypertension, and medication. Interviews were conducted in a secure place in the hospitals maintaining privacy after obtaining written consent of the participants.

#### Outcome variable

A structured, eight-item EQ-5D-3L tool was adapted to assess the HRQoL of the participants. The HRQoL records were self-reported and the tool was previously validated in different study settings prior to conduction of interviews [17]. The EQ-5D-3L (© 1990 EuroQol Group. EQ-5D™) questionnaire was developed during 1990 by the European investigators [20]. It applies to a diverse range of diseases and health conditions as it provides a self-reported visual analog scale (VAS) and a single index value for health status. This measure uses a scale assuming values between 0 and 1, with 0 indicating a health state that is equivalent to death and 1 indicating perfect health status [21]. This instrument has been used previously worldwide on different hypertensive populations [22, 23]. The health description system section records self-assessed health status according to five key dimensions: mobility, self-care, usual activities, pain/discomfort, and anxiety/depression. Each of the assessed dimensions is further split into three levels: no problem, some problems, and extreme problems. A total of 243 health conditions can be expressed by combining the different levels from each dimension. We used an adapted and validated Bengali version of the EQ-5D questionnaire to overcome language barriers. The translation of this instrument was undertaken independently from the EuroQol group and was validated for the Bangladeshi population and pre-tested in a small sample of 30 patients in two hospitals.

#### Independent variables

The study used the independent variables, mostly categorical, representing different socio-demographic characteristics. Ages were categorized as “18-29”, “30-39”, “40-49”, “50-59”, and “60 and above”, and the educational attainment of the respondents were categorized as “Not educated”, “Primary school”, “Secondary school”, “Higher secondary”, and “Bachelor and above”. Marital status was recorded with three levels namely “Married”, “Unmarried”, and “Separated or widowed” and employment status with “Not employed” and “Employed”. The total family income of individuals were categorized as “Less than USD238”, “USD238-<USD417”, “USD417-<USD595”, “USD595-< USD893”, “USD893-< USD1,190”, and “≥ USD1,190”. Information on gender and residence were also collected. The study included two dichotomous variables, family history of hypertension and medication use. Self-reported comorbidities entered different models as count variables. Anthropometric measurements of weight, height, were measured using standardized protocols and calibrated equipment and used for calculation of BMI. Systolic and diastolic blood pressures (BP) were measured twice using digital BP machines (Omron, SEM-1, Omron Corp., USA) at 10-minutes intervals and the averages of the two readings were used for this analysis. In addition to collecting systolic and diastolic BP data, other co-morbidities were recorded from the self-report during interviews, which were further confirmed by reviewing each participant’s medical records.

#### Comorbidity measurement

Comorbidity data were determined by self-reports by the participants initially through yes/no responses to the questions implying “Has a doctor ever diagnosed that you had...”. The trained interviewer also verified the reported chronic illnesses through checking previous medical records of the patients, and laboratory diagnosis reports as well as the medications list of the participants to ensure the accuracy and comprehensiveness of the data.

#### Ethical considerations

This study was approved by the Ethical Review Committee of Chittagong Medical College Hospital (CMC/PG/2020/27). Written informed consent, before the interview, was obtained from all participants. The objectives and procedures of the study were explained to the participants in their native language (Bengali).

#### Data analysis

A total of 1,963 hypertensive individuals were approached, of whom 1,912 respondents completed the full survey and thus constitute the final study sample. Descriptive analyses were carried out by comorbid and non-comorbid groups, where the comorbid group included participants who reported having at least one chronic condition. Those reporting no chronic morbidity other than hypertension constituted the non-comorbid group. Continuous variables were analyzed as means and standard deviations and the categorical variables as frequencies and proportions along with presenting 95% confidence intervals and chi-squared test. The two continuous variables named systolic BP and diastolic BP were found to be normally distributed. No specific model selection techniques were used in finalizing the equations of interest as followed by Zhang L et al (2017) and Zhang Y et al (2016). An ordered logit regression model was estimated for each of the five problem dimensions of EQ-5D measure namely mobility, self-care, usual activity, pain, and anxiety/depression. Ordered logit regression model was used because of the inherent ordering of the health dimension states that include “no problem”, “some problem”, and “extreme problem”. Finally, multiple linear regression models were used to estimate the ceteris paribus impact of comorbidity on HRQoL of hypertensive patients. For the robustness check, a simple linear regression model, with comorbidity as the only independent variable, was estimated. Then, the stability of the regression estimates was tested by step-wise addition of the socio-demographic variables and the health-related variables. The Variance Inflation Factors (VIFs) were calculated to check if multicollinearity exists in the data. All VIFs were found to be smaller than 10, indicating that multicollinearity may not be a problem. No specific model selection techniques were used in finalizing the equations of interest as followed by Zhang et al 2017 and Zhang et al 2016 [24, 25]. STATA/MP 14 (StataCorp LLC, Texas, USA) and GraphPad Prism (9.0, GraphPad Software, CA, USA) were used for the statistical analyses.

## Results

The respondents of this study belonged to the 18 – 98 years age group. The median age of the respondents was found to be 52 years. About one-third (33.3%) of the respondents were between 50 and 59 years old, and 56.2% were female (Table 1). A vast majority of participants (86.5%) were married. Approximately, 34% of respondents had a bachelor's education while 62% were not engaged in work. Half of the participants came from lower-to-middle income families, with family income ranging between USD238 to USD595. Most respondents (59%) lived in urban areas. The respondents were mostly overweight (43%) with BMI ranging from 23.0 to 27.5 kg/m^2^ and obese (27.1%) (Table 1). About 67.6% of the respondents had a family history of hypertension while 85.5% reported taking the antihypertensive medication regularly.

**Table 1:** Characteristics of the study sample by comorbidity status

	Total (*N*=1,912)	Without comorbidity (*N* =668)	With comorbidity (*N* =1,244)	*P*-value^1^
	*N* (%)	% [95% CI]	% [95% CI]	
***Socio-demographic characteristics***				
Age group (years)				<0.001
18-29	117 (6.2)	11.8 [9.6-14.5]	3.1 [2.2-4.2]	
30-39	168 (8.8)	13.2 [10.8-16]	6.4 [5.2-7.9]	
40-49	450 (23.5)	30.1 [26.7-33.7]	20 [17.9-22.3]	
50-59	637 (33.3)	26.8 [23.6-30.3]	36.8 [34.2-39.5]	
60 and above	540 (28.2)	18.1 [15.4-21.2]	33.7 [31.1-36.4]	
Gender				<0.05
Female	1075 (56.2)	59.4 [55.7-63.1]	54.5 [51.7-57.3]	
Male	837 (43.8)	40.6 [36.9-44.3]	45.5 [42.7-48.3]	
Education level				>0.05
Not educated	102 (5.2)	5.1 [3.7-7]	5.5 [4.3-6.9]	
Primary school	368 (19.3)	16.6 [14-19.6]	20.7 [18.5-23]	
Secondary School	412 (21.6)	21 [18-24.2]	21.9 [19.7-24.3]	
Higher Secondary	373 (19.5)	20.7 [17.7-23.9]	18.9 [16.8-21.2]	
Bachelor and above	657 (34.4)	36.7 [33.1-40.4]	33.1 [30.6-35.8]	
Marital status				<0.001
Married	1654 (86.5)	84.1 [81.2-86.7]	87.8 [85.8-89.5]	
Unmarried	100 (5.2)	9.3 [7.3-11.7]	3.1 [2.2-4.2]	
Separated or widowed	158 (8.3)	6.6 [4.9-8.7]	9.2 [7.7-10.9]	
Employment status				>0.05
Not employed	1189 (62.2)	59.9 [56.1-63.5]	63.4 [60.7-66.1]	
Employed	723 (37.8)	40.1 [36.5-43.9]	36.6 [33.9-39.3]	
Family income				>0.05
<USD238	365 (19.1)	19.8 [16.9-23]	18.7 [16.7-21]	
USD238-<USD417	489 (25.6)	24.9 [21.7-28.3]	26 [23.6-28.5]	
USD417-<USD595	477 (25.0)	26.2 [23-29.7]	24.3 [22-26.7]	
USD595-< USD893	342 (17.9)	15.9 [13.3-18.8]	19 [16.9-21.2]	
USD893-< USD1,190	148 (7.7)	7.8 [6-10.1]	7.7 [6.4-9.3]	
≥ USD1,190	91 (4.7)	5.5 [4-7.6]	4.3 [3.3-5.6]	
Residence				>0.05
Rural	778 (40.7)	40.4 [36.7-44.2]	40.8 [38.1-43.6]	
Urban	1134 (59.3)	59.6 [55.8-63.2]	59.2 [56.4-61.9]	
*** Health-related characteristics***				
Body Mass Index				>0.05
Underweight	76 (4.0)	3.7 [2.5-5.5]	4.1 [3.1-5.4]	
Normal	484 (25.3)	26.9 [23.7-30.4]	24.4 [22.1-26.9]	
Overweight	735 (38.4)	39.4 [35.7-43.1]	37.9 [35.3-40.7]	
Obese	617 (32.3)	29.9 [26.6-33.5]	33.5 [30.9-36.2]	
Systolic BP (mmHg), $\overline{X}$ (SD)	140.713 (14.5)	142.4 [141.4-143.5]	139.8 [139.0-140.6]	
Diastolic BP (mmHg), $\overline{X}$ (SD)	88.124 (11.6)	88.8 [87.9-89.7]	87.7 [87.1-88.4]	
Family history of hypertension				>0.05
No	619 (32.4)	33.2 [29.8-36.9]	31.9 [29.4-34.6]	
Yes	1293 (67.6)	66.8 [63.1-70.2]	68.1 [65.4-70.6]	
Hypertensive medication				<0.001
No	277 (14.5)	18.1 [15.4-21.2]	12.5 [10.8-14.5]	
Yes	1635 (85.5)	81.9 [78.8-84.6]	87.5 [85.5-89.2]	

*CI* Confidence interval*, BP Blood pressure, SD Standard deviation*

^*1*^*p values were analyzed using chi-square tests, and p<0.05 was considered statistically significant*

**Fig. 1:**
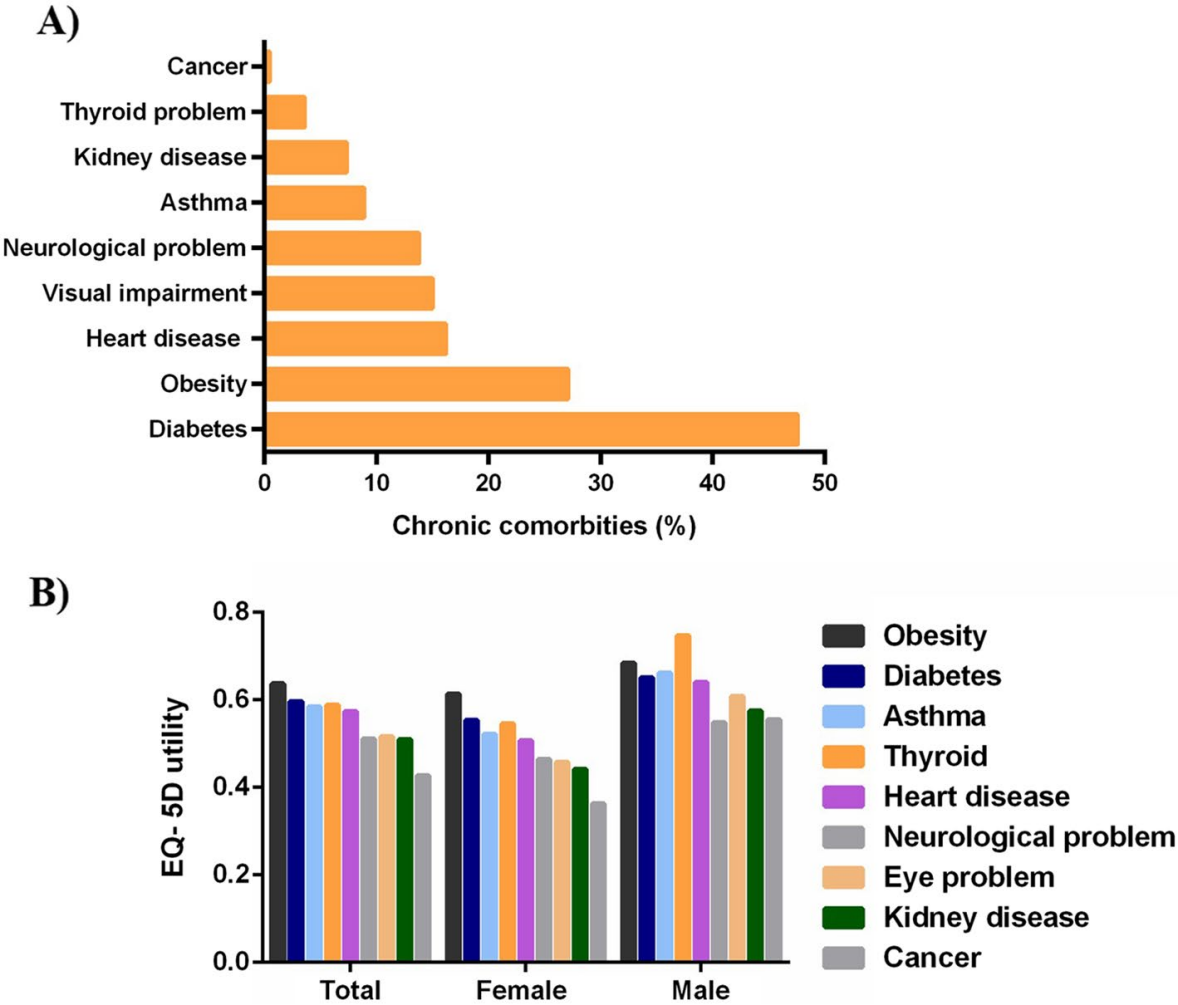
Frequency of various comorbidities among hypertension patients of Bangladesh. **A** Frequency of various comorbidities; **B** Mean value of EQ-5D utility by comorbidities

**Fig. 2:**
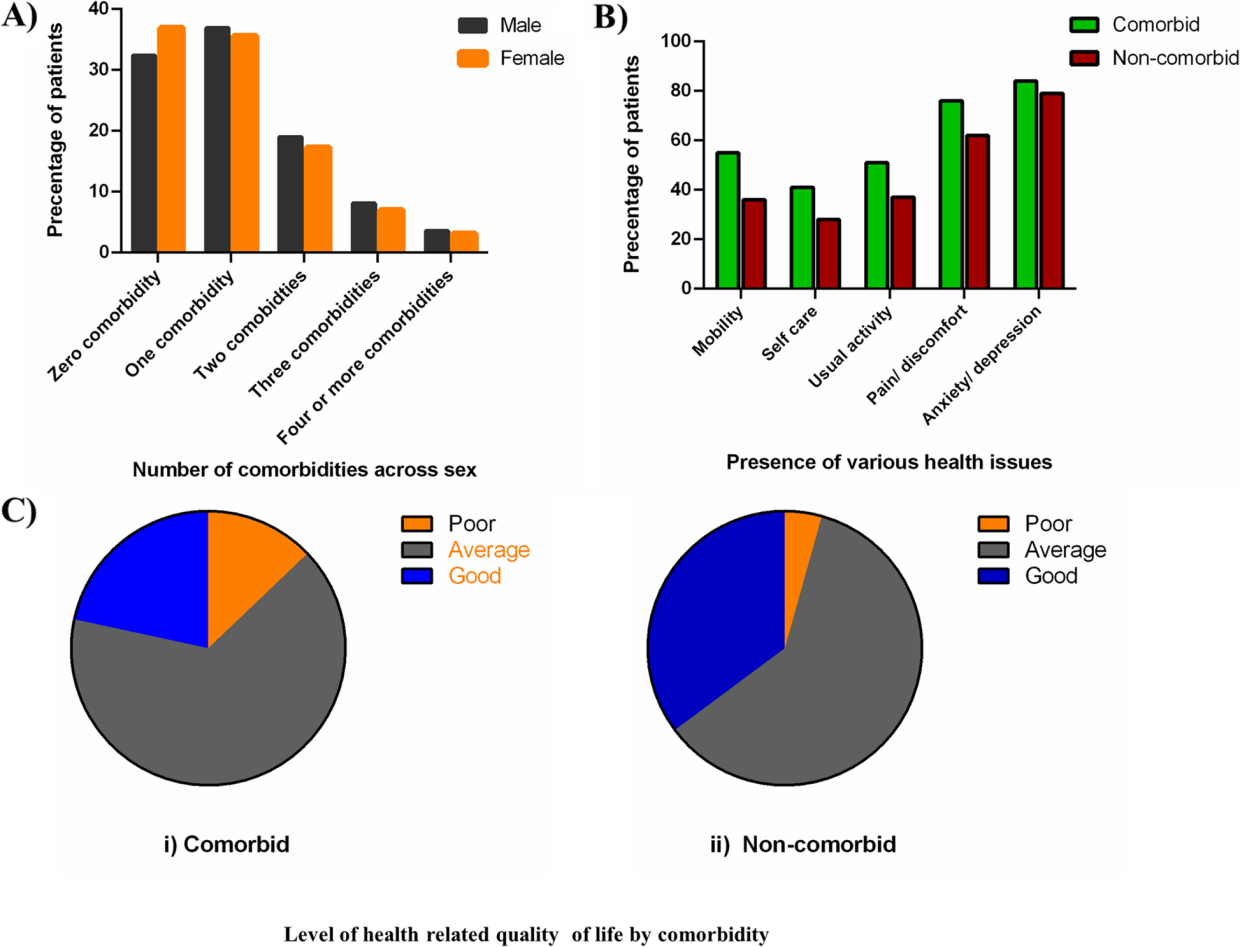
Frequency and effect of comorbidity on health-related quality: **A**) Comparison between number of comorbidities among male and female; **B**) HRQL among comorbid and non-comorbid patients. The EQ-5D scores were categorized as follows: “Poor”(EQ-5D ≤25^th^ percentile =.516), “Average” (EQ-5D >25^th^ percentile =.516 and ≤ 75^th^percentile =.796), “Good” (EQ-5D >75^th^ percentile =.796)

A total of 1,244 hypertensive patients (65%) reported having suffered from at least one chronic condition (mean=1.09, and SD=1.10). Among the respondents, 36.2% had single comorbidity, 18% had two, 7.5% had three, and 3.4% had four or more comorbid conditions. Males were found to have more comorbid conditions, showing higher percentages in all types of comorbidities, compared with females (Fig. 2A). However, the bivariate relationship between gender and number of comorbidities were found to be statistically insignificant. Compared to the participants with no comorbidities, a higher proportions of those with comorbidities reported that they suffered from the five problems associated with the health related quality of life (Fig. 2B). All chi-square tests, examining the association between comorbidity status and the problem dimensions, were found to be significant at levels less than 5%. Supplementary Table 1 provides a comparison between non-comorbid and comorbid respondents, based on three levels: no problem, some problem, and extreme problem of the five dimensions. Diabetes was found to be the most prevalent (47.6%) comorbid condition, while obesity being the second (32.3%), followed by heart disease (16.2%), visual impairment (15%), and neurological disease (13.8%) (Fig. 1A).

Table 2 shows the findings of the ordered logit models, which indicated a positive relationship between comorbidity and the probability of having physical problems. Compared with individuals without comorbidity, having one comorbidity, two comorbidities, three comorbidities, and four or more comorbidities were predicted to increase the log odds of being at a higher level of “mobility problem” by 1.42, 2.01, 3.51, and 3.67, respectively. The estimated models for self-care problem (OR: 1.39, 1.62, 2.72, and 2.35), usual activity problem (OR: 1.42, 1.71, 2.08, and 5.41), and pain (OR: 1.48, 1.85, 3.03, and 7.00) show comparable effects as well. Finally, patients with two, three, and four or more comorbidities, in comparison with those with no comorbidity, were predicted to have an increased probability of being at a higher level of anxiety/depression (OR: 1.13, 2.34, 2.05 and 11.17).

**Table 2:** Odds ratios from ordered logit estimates for the five dimensions of HRQOL

	mobility	Self-care	Usual activity	pain	anxiety
Age group (years)					
18-29 (reference)					
30-39	1.198	1.145	.886	1.822*	1.814
	(.448)	(.47)	(.321)	(.627)	(.699)
40-49	1.518	1.878	1.122	2.6***	1.719
	(.542)	(.734)	(.385)	(.867)	(.631)
50-59	1.937*	1.906*	1.428	2.824***	1.262
	(.692)	(.746)	(.49)	(.944)	(.461)
60 and above	3.899***	3.471***	2.404**	4.548***	1.106
	(1.408)	(1.36)	(.836)	(1.555)	(.409)
Gender					
Female (reference)					
Male	.601***	.816	.582***	.548***	.875
	(.086)	(.122)	(.083)	(.08)	(.136)
Education					
Not educated (reference)					
Primary school	.465***	.354***	.509***	.662	1.029
	(.12)	(.087)	(.129)	(.189)	(.296)
Secondary School	.592**	.38***	.514**	.795	1.055
	(.157)	(.096)	(.134)	(.231)	(.312)
Higher Secondary	.519**	.419***	.546**	.721	.881
	(.143)	(.111)	(.148)	(.217)	(.271)
Bachelor and above	.562**	.287***	.521**	.788	.714
	(.154)	(.077)	(.141)	(.236)	(.217)
Marital status					
Married (reference)					
Unmarried	.842	1.385	.66	1.4	.938
	(.325)	(.566)	(.249)	(.495)	(.361)
Separated or widowed	1.911***	1.599**	1.402*	1.38	1.473*
	(.391)	(.306)	(.278)	(.314)	(.34)
Employment status					
Not employed (reference)					
Employed	.707**	.638***	.558***	.754**	1.132
	(.099)	(.094)	(.078)	(.108)	(.176)
Family income					
<USD238 (reference)					
USD238-<USD417	.909	.744*	.784	1.21	.877
	(.138)	(.115)	(.118)	(.197)	(.154)
USD417-<USD595	.885	.935	.845	.889	.939
	(.139)	(.148)	(.13)	(.147)	(.169)
USD595-< USD893	.84	.939	.706**	.715*	.671**
	(.143)	(.163)	(.12)	(.127)	(.13)
USD893-< USD1,190	.984	.79	.658*	.808	.941
	(.216)	(.182)	(.146)	(.183)	(.237)
≥ USD1,190	.45***	.47**	.531**	.87	.515**
	(.125)	(.147)	(.144)	(.235)	(.145)
Body Mass Index					
Underweight (reference)					
Normal	.657	.668	.637*	.965	.79
	(.173)	(.171)	(.166)	(.265)	(.243)
Overweight	.532**	.52***	.446***	1.065	.797
	(.138)	(.132)	(.114)	(.288)	(.241)
Obese	.614*	.495***	.464***	1.077	.737
	(.162)	(.128)	(.121)	(.297)	(.227)
Systolic BP (mmHg)	.993*	.996	.998	.988***	.989**
	(.004)	(.004)	(.004)	(.004)	(.004)
Diastolic BP (mmHg)	1.015***	1.018***	1.017***	1.011**	1.01*
	(.005)	(.005)	(.005)	(.005)	(.006)
Family history of hypertension					
No (reference)					
Yes	1.133	1.056	1.015	.926	1.431***
	(.125)	(.12)	(.111)	(.106)	(.176)
Hypertensive medication					
No (reference)					
Yes	1.259	1.475**	1.148	.929	.902
	(.196)	(.246)	(.177)	(.148)	(.157)
Comorbidity					
No comorbidity (reference)					
One comorbidity	1.421***	1.391***	1.419***	1.478***	1.126
	(.169)	(.175)	(.169)	(.178)	(.147)
Two comorbidities	2.012***	1.622***	1.713***	1.852***	2.342***
	(.293)	(.246)	(.25)	(.288)	(.419)
Three comorbidities	3.512***	2.723***	2.081***	3.029***	2.047***
	(.727)	(.55)	(.417)	(.697)	(.499)
Four or more comorbidities	3.668***	2.352***	5.413***	7.005***	11.165***
	(1.08)	(.676)	(1.658)	(2.374)	(3.532)
Residence					
Rural (reference)					
Urban	.873	.693***	.813**	.876	.971
	(.092)	(.075)	(.086)	(.097)	(.117)
Cut1: constant	1.223	2.428	1.061	.294	.12***
	(.882)	(1.79)	(.755)	(.219)	(.097)
Cut2: constant	86.375***	75.009***	55.617***	33.088***	15.34***
	(63.548)	(56.148)	(40.262)	(24.879)	(12.427)
Observations	1912	1912	1912	1912	1912
Pseudo R^2^	.111	.106	.105	.08	.057

*Standard errors are in parentheses*

^***^ p<.01, ** p<.05, * p<.1

The average EQ-5D scores for patients with reported chronic conditions are shown in Fig. 1B. The participants with obesity had the highest mean utility (0.64), followed by the diabetic patients (0.59) and those suffering from thyroid (0.58). The mean utility was found to be the lowest for those who were inflicted with cancer. For females with chronic conditions, the mean EQ-5D scores were lower (0.61) than their male counterparts (0.68). The results from a two sample *t* test, investigating the difference between the means of the EQ-5D scores for the two groups of male and female, exhibited statistical significance (p<0.01). Respondents with comorbidity were found to have more likelihood of reporting poor health quality than patients with no comorbidities (12.9% vs. 4.3%) (Fig. 2B). Table 3 presents the step-by-step estimated effects of the various levels of comorbidities in comparison with the reference category of no comorbidity on health-related quality of life. The equation in column 1 was unadjusted, using comorbidity as the only independent variable. The second equation is controlled for the socio-demographic variables and the final model is the complete model, controlling for both socio-demographic and health-related variables. The OLS regression results from all three models were quite consistent in magnitude and sign. All coefficients were statistically significant at the 1% level. The estimate was highly statistically significant and maintained almost identical magnitudes in the adjusted models as well, indicating that the estimated effects were insensitive to changes in regression specification. When compared with non-comorbid individuals, the health related quality of life for those having one comorbid condition diminished by 0.047. The EQ-5D score for respondents with two, three, and four or more comorbidities were found to decrease by 0.098, 0.168, 0.308, respectively, in comparison with non-comorbid respondents.

**Table 3:** Effects of comorbidities on health care related quality of life

	Model l	Model 2	Model 3
Comorbidity			
No comorbidity (reference)			
One comorbidity	-.057***	-.048***	-.047***
	(.013)	(.012)	(.012)
Two comorbidities	-.121***	-.10***	-.098***
	(.015)	(.015)	(.015)
Three comorbidities	-.188***	-.172***	-.168***
	(.021)	(.021)	(.021)
Four or more comorbidities	-.347***	-.311***	-.308***
	(.03)	(.03)	(.03)
Constant	.708***	.614***	.569***
	(.009)	(.041)	(.073)
Observations	1912	1912	1912
Adjusted R-squared	.097	.185	.186

*Standard errors are in parentheses*

^***^ p<.01, ** p<.05, * p<.1

*Model 1 is unadjusted, model 2 is adjusted for socio-demographic characteristics, and Model 3 is adjusted for both socio-demographic and health-related characteristics. The table reports only the coefficients of interest*

## Discussion

This study aimed to explore the HRQoL of the hypertensive patients of Bangladesh attributing the presence of other comorbidities and socio-demographic correlates. HRQoL was found to be inversely proportional with the increased number of comorbidities caused by decreased HRQoL of the hypertensive patients. As compared to patients with no comorbidity, an unambiguously decreasing trend in HRQoL utility score was observed in patients with one or more comorbidities. The finding aligns with the results of a study conducted in the Republic of Ireland, which presented that the presence of multiple comorbidities significantly reduces HRQoL [26]. Furthermore, compared to the individuals without any comorbidity, individuals having one or more comorbidities had significantly higher odds of having problems related to mobility, self-care, usual activity, pain, and anxiety/depression. This finding is particularly important underscoring the immense need for prevention and management of multi-morbidity among patients with a chronic condition like hypertension.

The most prevalent comorbidity in the study sample was found to be diabetes (found in almost half of the study participants), followed by obesity in nearly one-third of the patients with hypertension. Obesity and diabetes were also found to be the main contributors to poor HRQoL among patients with hypertension in Italy and Japan. It was reported widely that presence of metabolic syndrome including diabetes mellitus, obesity and hypertension had a negative influence on HRQoL in various populations [27, 28]. However, one previous study differed in terms of showing a similar association [29].

All the five health quality indicators such as mobility, self-care, usual activity, pain/discomfort, and anxiety/depression were found to be higher among hypertensive patients with comorbidities than the non-comorbid participants (Supplementary Table 1). Anxiety and/or depression were most commonly reported by more than three fourth of the hypertensive patients in this study followed by pain/discomfort and mobility problems. A substantially high proportion of psychological and mental health problems was reported with hypertensive patients in Africa [30]. Mental health issues are generally underestimated in many contexts including in Bangladesh. Addressing mental health challenges is important, to offer counseling and other psychosocial evaluation and support to individuals. Similar mental health counseling routines are also required by patients who suffer from chronic illnesses including hypertension. Significantly higher odds of having high levels of mobility, self-care, regular activity and pain were found among the cohort aged 60 years or more compared to the 18-29 years group. The highest odds in the 60 years or more age group were associated with mobility problems. Previous studies have defined hypertension (HTN) as an age-related chronic condition [31, 32]. McDaid et al, 2013 reported that the elderly hypertensive people (aged 50 years or more) perceived poor HRQoL [26]. Compared to females, males were significantly less likely to have mobility, pain and routine activity problems but this trend was not statistically significant for anxiety/depression and self-care dimensions [33]. Noteworthy to mention, lower levels of HRQoL were perceived in highly educated patients for mobility, self care and daily activity. Individuals with higher education are often found to hold important job positions. Pressure of work, multiple responsibilities and personal goals can make it difficult for them to prioritize self-care, leading to stress and burnout. Subsequent impact of the aforementioned factors may also be observed on their HRQoL. Compared to the low-income group, hypertensive patients from high income groups were significantly less likely to have mobility, regular activity and anxiety/depression problems. This finding supports the findings of an earlier research, which stated that unemployment and low socioeconomic status are associated with poor HRQoL [34, 35]. Patients with high diastolic blood pressure had significantly higher odds of having all the five health problems, compared to those who had high systolic BP. Compared to the unmarried patients, those who were divorced or separated had higher odds of having all five HRQoL related issues.

The average EQ-5D scores of the patients having chronic conditions are depicted in Fig. 1B. The overall highest mean utility (0.64) was observed in obese patients, followed by the diabetic (0.59) and disordered thyroid (0.58) patients and these findings are same for the female respondents. In contrast, the highest mean utility for male hypertensive patients was found in those having thyroid diseases, followed by obese, asthmatic and diabetic patients. The worst contributing factor of HRQoL among the study population and the women cohort was found to be cancer based on lowest overall mean utility. However; for men, the worst contributing factor was neurological problems. Least contributing comorbid conditions for women were obesity, diabetes and thyroid disorder whereas in men least contributing factors were thyroid diseases, followed by obesity, asthma and diabetes. According to Hay, 2016; gender difference should be taken into account to find out the mechanism behind the HRQoL affecting physical changes experienced by the hypertensive patients [36]. Based on EQ-5D scores, respondents with comorbidity had perceived level of poor health quality than non-comorbid patients (12.9% vs. 4.3%) (Fig. 2C).

This study has several implications. First, EQ-5D-3L has rarely been used in Bangladesh to evaluate the HRQoL of hypertensive patients. Nine types of hypertension-related comorbidities were addressed to assess HRQoL using the EQ-5D-3L. Second, local settings and culture were taken into account to generate EQ-5D utility; which removed the possibility of biases due to value set from other countries [37] or other cultural settings [38, 39]. Third, 1,963 hypertensive patients participated in this survey making this research one of the most significant population-based studies in Bangladesh to evaluate the impact of HTN on HRQoL. Fourth, hypertension has been reported as the most common non-communicable disease (NCD) in Bangladesh found in a nationwide survey with a prevalence of 21.0% (25.2% in urban areas) [40]. Lastly, this study aided us in understanding the condition of the hypertensive patient’s HRQoL in Bangladesh through an international standardized assessment tool due to adoption of EQ-5D-3L. Robust analyses using best-fit regression models were used to estimate the impact of hypertension and its related comorbidities on HRQoL adjusted according to the participants’ socio-demographic parameters along with comparison among them.

The present study, although it presented multiple positive outcomes, was not devoid of limitations. Firstly, there was skewness in the distribution of the characteristics of the patients towards elderly people. Secondly, hypertension was not differentiated according to the terms of types, stages, or severities. Thirdly, as it was a cross-sectional study, the results only provide associations and not causation. While EQ-5D scores calculated in multiple time periods would have provided a clear trend of changes in the HRQoL for the patients with hypertension, lack of funding was a pitfall during the collection of required panel data. For HRQoL it is very important to have repeated measurements, as HRQoL is a measure of change over time. The changes of scores over time are of importance. But the HRQoL could not be assessed at different time points in this study. Fourth, the experience from the pilot study impeded us from using the originally intended EQ-5D-5L instrument in the main survey. The questionnaire used in the pilot survey included all five levels of EQ-5D. However, we found a significant percentage of respondents found it difficult to differentiate between “some problem” and “moderate problem”, then we merged them as “some problem”. We experienced the same issue in the case of “severe problem” and “extreme problem”, again we merged these two levels as “severe problem”. Finally, selection bias could be a possibility because a convenience sampling technique was used due to the lack of a sampling frame for the hypertensive patients in Bangladesh.

## Conclusion

This study is the first in Bangladesh that used EQ-5D-3L on a substantial population-based sample of hypertensive patients to assess HRQoL. Hypertensive patients who were males, elderly, separated/ widowed, highly educated, being in the working-class, and with any comorbidity had lower perceived level of HRQoL. The involvement of psychiatrists, counseling psychologists, or mental health counselors in the management of hypertension should be considered with utmost importance due to the significant burden of depression and/or anxiety among hypertensive patients. This study can provide considerable insights to healthcare and policymakers to remodel the improved and effective hypertensive care in the country addressing the HRQoL. An additional longitudinal prospective study is warranted to confirm the trend and to study the causal relationship between hypertension (±comorbidity) and HRQoL.

## Supplementary Information

**Additional file 1: Table 1.** Prevalence of the problems considered in the HRQoL measure by comorbidity status.

## Data Availability

The data collected during this study will be available from the corresponding author upon reasonable request. Considering the ethical and confidentiality issue, it will be kept restricted.
