# Supplementary material for: Association between comorbidity and health-related quality of life in a hypertensive population: a hospital-based study in Bangladesh
Source: BMC Public Health. 2022 Jan 26;22:181. doi: 10.1186/s12889-022-12562-w (PMC8793199; doi:10.1186/s12889-022-12562-w)
Supplement: Supplementary file 1 — Additional file 1: Table 1. Prevalence of the problems considered in the HRQoL measure by comorbidity status. [file 12889_2022_12562_MOESM1_ESM.docx]

**Supplementary file**

**Supplementary Table-1**

| **Table-1** Prevalence of the problems considered in the HRQoL measure by comorbidity status | | | |
| --- | --- | --- | --- |
| **Dimensions of EQ-5D-3L** | **Grading/ Extent** | **Non-comorbid (%)** | **Comorbid (%)** |
| Mobility | No | 63.62 | 45.66 |
|  | Some | 35.93 | 51.45 |
|  | Extreme | 0.45 | 2.89 |
| Self-care | No | 72.01 | 59.24 |
|  | Some | 27.10 | 37.30 |
|  | Extreme | 0.90 | 3.46 |
| Usual activity | No | 62.87 | 48.63 |
|  | Some | 36.23 | 48.15 |
|  | Extreme | 0.90 | 3.22 |
| Pain | No | 37.72 | 24.12 |
|  | Some | 61.53 | 71.22 |
|  | Extreme | 0.75 | 4.66 |
| Anxiety | No | 20.66 | 16.40 |
|  | Some | 77.25 | 77.09 |
|  | Extreme | 2.10 | 6.51 |
